# Home and Office Treatment of Inebriety

**Published:** 1912-12

**Authors:** T. D. Crothers

**Affiliations:** Superintendent Walnut Lodge Hospital, Hartford, Conn.


### Home and Office Treatment of Inebriety

By T. D. CROTHERS, M. D.,
Superintendent Walnut Lodge Hospital,
Hartford, Conn.

WITHIN a comparatively recent period, it has been noted that physicians are called on, with increasing frequency, to treat persons who are intoxicated or suffering from some disorder that has followed the excessive use of spirits.

Often these persons are from the best families and among those who are the warmest patrons of the physician. They are suffering from delusional states, have defects of vision and hearing and other conditions which they realize are the results from spirit drinking, and turn to the physician with pathetic eagerness for help. They are alarmed, mortified and excited and this is increased by the anxiety of relatives and friends.

The physician is often distressed at the impotency of his efforts and is in doubt as to the best methods and means to be used. If he gives opium or its alkaloids, there will be stupor, following delirium, and not unfrequently narcotism with the possibility of fatal termination.

When this occurs his anxiety is increased with the thought that the narcotic was the most active cause. If he tries emetics and purgatives, unexpected results may follow and he is discredited in the mind of the patient, as well as in his own judgment.

If he tries placebos or remedies laid down in the book, there is the same uncertainty and often failure in most unexpected ways. Later in disgust he tacitly agrees to have the patient try the gold cures or go to some secret quack institution.

Finally the patient returns restored and is buoyed up with the delusional confidence that he is cured, and is emphatic in his praise of the value of these means. The physician is certain that no new remedies are employed; that the managers of these places are not medical men of repute, and scientific attainments, and do not possess anything in the nature of drugs with which other physicians are not familiar.

Now when members of old families who have been devoted friends obtain relief from such doubtful sources and by secret means, promoted by charlatan methods, a feeling of deep regret pervades the physician's mind, that he could not have done as much with his knowledge and training. This is the common experience of every general practitioner.

Another fact is growing more and more apparent, namely the failure of clergymen, philanthropists and reformers to render aid to these poor unfortunates; also the harshness of legal methods by fines and imprisonments. Clearly the physical side of the subject is unrecognized and as a result the conditions are intensified and the persons are made more and more incurable.

The possibility of a practical home treatment is a reality to a far greater extent and is becoming practically recognized in various ways. Thus in the large cities, hotels find it practical to employ physicians, their principal work being to treat persons in the acute stage of intoxication.

The early practice of physicians brings them in contact with these cases more than ever. They are sent for on the supposition that they possess more acute knowledge of the conditions, and that their personal knowledge of the patient will enable them to confine their attentions to the present conditions.

The older physicians often retain the old time prejudices and theories that these sufferers from drink excess are moral weaklings, that they are simply reckless and should suffer, that they need moral reprimands and appeals to that side, rather than drugs and physical measures.

It is this attitude that has driven patients away to the charlatan homes who could and should have been treated by the family physician.

Some of the facts which are not clearly understood may be grouped as follows. A person, stuporous or delirious with an alcoholic breath and a history of having used spirits to great excess is suffering from a serious pathological condition. While alcohol may have been the exciting cause, there may be present numerous complications such as blows on the head, sun strokes, or some sudden mental shock, disturbing and paralyzing the functional activities of the brain, which are exciting and contributing causes.

A man who is drinking at intervals receives a severe injury and very soon afterwards he becomes intoxicated, manifests strange symptoms. Evidently the alcohol is not the chief cause. Intoxication is always a pathological toxemia, palsy, congestion of certain brain centers, also a culmination of continued shocks from toxemias and cumulative organic degenerations with spasmodic alterations of nerve energy. These acute symptoms may pass away, but the injury which follows from them is not removed or repaired at once.

A very curious question was asked in a certain circle, viz: How often could a person become intoxicated, meaning stupid or delirious from the effects of spirits and apparently recover? One author claimed that he had noted several hundred attacks of this kind in different persons. Another claimed it possible to have a much larger number of toxemias.

In my experience I think from sixty to one hundred times is about the limit, and in most cases long before this, permanent alterations begin and the person is insane and demented or dies from some acute disease. Not unfrequently the drinking man has long periods of sobriety between the drink excesses. During this time a certain degree of restoration takes place, hence he might live a great many years and be intoxicated at long intervals, but it is certain that a man who drinks to the toxic stage frequently, dies early or becomes insane.

The first question in the practical treatment of such persons is to determine whether the person is a chronic drinker, that is one who has drank for years even in so-called moderation, or occasionally to excess? Is the patient a periodic drinker with periods of distinct abstinence? Is he a continuous drinker, taking wine at the table or beer at short intervals? If a periodic drinker the tendency of the case is to recover. The paroxysm dies out and the desire for spirits disappears, and the treatment required is to encourage this condition as far as possible and not complicate it or retard the progress.

If the person is a continuous drinker a very complex condition of poisoning exists. General degenerations and perversions of all the functions are present. Toxins from without and within are present and the most careful studious examination must be made.

Some acute conditions or some symptoms out of the ordinary appear before the physician is called and it is safe to assume that the conditions are far more serious than they appear on the surface. The person is maniacal and delusional or stupid and demented to some degree, often not apparent to casual observation. If the person is a periodic drinker and the usual time of drinking is nearly ended, powerful elimination may be resorted to. If gastritis is not present large doses of ipecac may be given or apomorphia, particularly if the patient has been insomniac for some time. If the bowels have been constipated, a drastic cathartic, such as croton oil has a very good effect. The three days quack treatment, depends entirely on elimination by the bowels, vomiting and profuse diaphoretic action. This continued vigorously for 24 hours or 48 hours, associated with hot baths, eliminates the toxins and is such a severe drain on the system as to change the activities of the entire organism. This is rational treatment for a certain number of cases. The charlatan application is used in every case indiscriminately up to the point of profound collapse, and associating with it suggestion and the dominance of an idea that he can never drink again and is thoroughly cured.

Hot baths and fomentations over the body are the safest and most effective measures to produce eliminations of the toxins. In one of the stories of Dickens a large number of voters were so much intoxicated as to be unable to exercise that function. They were taken to the town pump and after being thoroughly drenched with water, recovered or were made sober by it, and then were able to cast their ballots. This was a very good practice.

All institutions that give scientific treatment for these toxic cases, find water in the form of either hot or cold douches or packs a most valuable measure. This can be substituted in private homes by vigorous rubbings and hot applications. If the patient is in a delirious condition and resents all efforts to do anything for him apomorphia by the needle in 1/10 of a grain doses is the most powerful relaxant which can be used.

Sometimes extreme nausea follows, and usually narcotism. Smaller doses of from 1/30 to 1/50 of a grain, given at intervals of two or three hours, change the tension of the mind and body and unless there is some organic disease of the heart or arteries, the results are very satisfactory. If the case is simply stuporous, tincture of ipecac will be a simpler eliminative; that and salines would be less likely to be followed by any drug effects. Morphine and chloral are dangerous. The later always increases the delirium for a time before its anaesthetic effect is produced. If the patient is an alcoholic, the chloral is dangerous, because the drug being one of the alcohols may act on the vital centers lowering them beyond the point of recovery or at all events produce additional disturbances of irritation and congestion.

The morphia is also uncertain because of the possible unknown narcotic action. Eighth and quarter grains are sometimes stimulating, and then suddenly center in pronounced depressive states.

In private practice it is always unsafe to give narcotics without some personal knowledge of the patient, particularly of the conditions present. Morphia may act as an additional toxaemiac, checking elimination and indirectly intensifying the very condition which it is supposed to remove. When given it should be very carefully watched, until its possible effects are beyond question.

Not unfrequently sudden death takes place in a person intoxicated who has been given a dose of morphia. A man at a banquet became profoundly intoxicated; was taken into a side room in a state of muttering delirium and after a quarter of a grain of morphia, died. There was no question but the morphia precipitated the final condition.

Apomorphia is far safer, although equally powerful, because of its irritant effects. A continuous drinker awoke one night with hemiplegia. The physician increased the dose of spirits with an addition of strychnine and morphia. The man died in a short time. Sharp eliminatives, cathartics and removal of the spirits would have taken away the active exciting causes and given the man a better chance to recover.

The treatment of acute conditions in home and office practice calls for subsidence or relief from the positive toxemiac conditions present. Thus acute gastritis may be relieved by stomach lavage with hot and cold water fomentations on the outside and along the spine, combined with rest and the correction of acid or alkaline conditions.

A very foolish theory that spirits will relieve this condition if combined with eggs and milk, also other substances is still urged. Chronic inebriates often treat themselves by increased or reduced doses of spirits or wines, beers and other alcohols, and claim great results from them. The delusional theory that alcohol is a stimulant, based on the fact that it raises the heart's action, explains many of the difficulties of the treatment of such persons.

The apparent stimulant action is simply an irritant, followed by depression which quickly lowers all the functional activities of the organs to the narcotic stage. Hence the man suffering from the effects of alcohol is depressed, and anaesthetized. He may have been suffering from auto intoxications and metabolic changes of nutrition, before spirits were used. Then alcohol adds additional anaesthesia and depression.

The man who becomes profoundly intoxicated at the banquet is suffering from several states of poison. The alcohol has lowered the nutritional activities and diminished the heart's capacity to propel the blood. Foods and fluids are fermenting and dilating the stomach, and this is pressing on the heart, still further diminishing its power. There is vasomotor paralysis, defective oxidization, accumulation of salts, acids of toxins, with a tendency to localized spasms in the brain centers.

Elimination from the stomach, bowels and through the skin by the simplest methods and means is called for. What damage is done to the neuronic centers and how far the heart will react is unknown. The subsidence of the acute symptoms may reveal other grave conditions, demanding immediate attention.

In the person who has drunk continuously in so-called moderation and seldom seemed to show the effect of spirits, there are always defective nutrition, depressed vitality and localized inflammatory conditions, following congestion and the corroding effects of alcohol.

His own estimation of his condition should never be relied upon. Many persons of this character who have used spirits for years in some form or other complain of what is called rheumatism which is literally neuritis, and also of stomach troubles. They have congested faces which must be recognized as an indication of the circulation of blood vessels of the brain.

Obscure symptoms that point to grave organic degenerations, not unfrequently conceal the real cause and prevent the use of active measures that would otherwise be used. Insomnia, muscular trembling and palsy, local or general appear. Persons of this character go to specialists in the large cities and after most elaborate diagnosis and treatment, fail to secure any benefit.

If the specialist or physician uses spirits personally, he will underrate the effects of alcohol, most naturally, and often overlook its influence as a factor in the case under treatment. Many illustrations of this are seen. Thus a very good physician of this class failed in his treatment of a family that was accustomed to use wine and spirits both as a medicine and at the table as a beverage.

A younger physician insisted on spirits being withdrawn, and impressed his views on the family so sharply that he superseded the older one.

In the home and office treatment all cases that are complex with any kind of a history of the use of alcohol should be regarded as possibly due to alcohol, and total abstinence should be the first step. Then remedies addressed to elimination and correction of possible toxemic states should be used.

In alcoholic delirium and exalted states apparently due to spirits hydrtherapeutic measures are the safest. Muscular delirium can often be treated by an opportunity of working it off in the open air. In a case of delirium tremens I was called to see, a powerful man was strapped down on a bed. He was struggling severely. He was removed and sent out walking between two men, and in the course of half an hour he returned, exhausted and made a good recovery.

Apomorphia was given him in very small doses and this broke up his delusional condition. In another case of delirium all drugs were removed and the patient was placed in a warm bath and kept there for two hours. Then relaxation took place. It is much safer to treat cases of delirium by hydrtherapeutic measures and free exercise with the necessary eliminatives than by narcotics. The old time methods of a straight jacket with restraint, should be avoided, unless absolutely necessary.

There are always a certain number of persons who while having delirium and delusions are considered dangerous. They should be sent to an institution or placed under the care of persons who are familiar with this class of disorders. Not unfrequently persons in this unsettled mental state come under my care, and on recovery go back to the physician and are treated with great success. There is no thought of final cure or claims of new remedies with new effects, but simply states of disease that require removal. The family physician of all others is the best fitted to give the after care and treatment.

Often such persons are narcotized by the physician and develop very grave symptoms, suggesting some form of insanity. They come under my care and are practically drug victims, suffering from bad treatment on a starved and poisoned organism. There is so much sentiment and so many mistaken theories and deceptive efforts to conceal the real cause that the physician is often very seriously prevented from doing what his own judgment would suggest.

Frequently gormandizing and irregularities of living precede and follow the use of spirits and complicate the conditions so seriously that institutional treatment is required. Here the patient can be restored to a normal condition and in a measure taught how to live after, and then with the help of the family physician make a good recovery.

There is something very absurd in the statement that persons who have drunk to excess can in four weeks recover permanently by any form of treatment. Their statements of final restoration and cure can only be verified after a long period of years.

If after a short treatment in institutions they could go back under the care of the physician and retain him as a permanent adviser and counsellor and consult him often, there would be much promise for the future.

The best experience covering a period of nearly forty years in the active care of these persons, fully confirms this. Like the tuberculosis cases, a few months residence in a sanitorium gives them strength and capacity to overcome the disease and with it a knowledge of how to continue the treatment. Then the care of the family physician added to this makes the permanence of restoration very pronounced.

The office treatment of inebriates is unsatisfactory and often very annoying in the opinion of many physicians. Doctors having extensive practice, especially in large towns and cities are frequently consulted by persons under the influence of spirits. It is a problem what to do for them. A physician in Worcester prescribed a quarter of a grain of morphia and the man was found dead a few hours later. In another instance chloral was prescribed. The man went to a saloon, drank more and committed a homicide.

There is so much uncertainty and actual peril in office treatment of persons of this class that many physicians refuse advice or give medicines under these circumstances. I have found a concentrated solution of quassia to be the most effective of all the remedies. An infusion of quassia boiled down once or twice to obtain double the strength may be given to a drinking man with strict injunctions to use a half an ounce every two hours, through the day. He is told that he may drink as usual, but under no circumstances must he neglect to use the medicine. In a very short time a species of quassia poisoning comes on, and all forms of spirits become intensely disgusting and really repelling.

The patient's mind is startled and pleased to think that he can't drink spirits and while the quassia is very unpleasant, he continues its use. The thought grows on his mind that he probably can never drink spirits again, and he returns to the physician with the deepest gratitude.

This is the opportunity for the physician to get possession of him and begin a course of treatment that in many cases brings about final restoration. Cinchona bark will do the same thing and was some years ago exploited as a specific remedy, but quassia is simpler and seems not to complicate any of the function of the body, other than to irritate the stomach, provoking an increased appetite.

An illustrative case is worthy of note. In a Massachusetts town a delirious haggard-looking man appealed to a doctor to help him, saying he had drunk for days and was in the deepest despair. He had called on other physicians and been turned away. Some of them gave him money to procure more spirits, others treated him harshly. He declared he wanted to stop the use of spirits, that he was literally crazy and had that morning tried to kill his wife and himself, and end his existence.

The doctor gave him a large dose of apomorphia and sent him out to the stable under the care of the hostler. He vomited freely and went to sleep. A few hours afterwards he awoke and was given another dose. This relaxed him still more. Then he was given a warm shower and a large dose of quassia. This was kept up for nearly two days in the stable, then the man was sent home.

He returned to the physician every day taking quassia, together with other tonics and made a very good recovery. Eventually he became a wealthy man, and was one of the warmest friends of the physician. This showed the possibility of practical treatment. In periodic drinkers the return of the paroxysm is often predicted by the patient and his family in certain erratic symptoms of conduct and thought.

The physician is appealed to, to prevent the paroxysm. Each case is a problem of itself. Not unfrequently morphia is given and the paroxysm is broken up, but the patient discovers a means which he will use on future occasions with the danger of a morphine addiction. It should be remembered that all these drink neurotic cases turn very quickly to drug addictions and, hence narcotic drugs should be used with great care and secrecy. A safe combination which can be used on the approach of the drink paroxysm is nitrate of strychnine and sulphate of atrophia, 1/60 of the former to 1/100 of the later. This can be given every three hours. If with this, calomel purgatives are given and baths, often the paroxysm may be averted.

Many persons find this combination a very satisfactory one and unless there is some peculiar sensitiveness to these drugs, it can be continued for some time. Both atrophia and strychnine are the basis for all the gold cures, with additions of drugs, which are of less value.

In a moderate drinker, after the subsidence of the drink craze, a combination of this kind with 1/200 of a grain of atropia may be given three or four times a day, and should any signs of poisoning appear, it should be stopped for a week or two, and then resumed in smaller doses.

Phosphate of soda is another remedy which in certain cases, particularly where there is a tendency to acidemia, is followed by the very best effects. In many of the spirit neurotics, insomnia is a frequent after symptom. Sometimes this is successfully treated by baths, but where these are not available, some of the mild narcotics can be used, namely lupulin or hops. I find that the ordinary infusion of hops acts well. The preparations on the market are frequently old and inert. Valerian is another excellent remedy. As a tincture it is not always safe. There is alcohol enough in it to keep up an irritation which will sometimes break out in the drink paroxysm.

Bromides may be used, particularly bromide of sodium. The insomnia often passes away and is followed my nutrient disturbances and debility, frequent exhaustion and nervousness. These require special remedies to be given for short periods, and always with the understanding that the patient is supersensitive to narcotic drugs, and may become a drug taker.

States of what are called neurasthenia and psychaesthenia follow after spirits have been abandoned, and the family physician who may know of the previous addiction should never minimize symptoms of this kind. Persons who come for treatment of these complex nerve troubles, together with nutrient disturbances, who give a history of having used spirits at some previous time require great discretion and judgment, with careful prognosis.

They resemble the obscure symptoms that frequently follow, years afterwards in cases of syphilis which disappear after mercurial treatment. These symptoms give way readily to eliminative measures and certain bitter compounds like the strychnine and atropia or certain mild narcotics. In the opinion of many physicians, phosphate of soda is a remedy of the greatest value. At all events it can be used with safety.

Cirrhosis is a very common condition following the use of spirits. The high tension arterial measurements indicate its presence and its organic character is assured by the persistency of these symptoms. The iodides may be given for this condition, and arsenic preparations. All such patients should be impressed with the necessity of frequent consultation and the great difficulty of permanent help.

A large proportion of the cerebral hemorrhages can be traced to alcohol and cirrhotic arteries. The specific action of alcohol paralyzing the vaso-motor nerves and producing excessive fibous deposits in the system, and about the arteries provokes hemorrhage. The physician by wise counsel may prevent this condition and greatly lengthen life.

The moderate and excessive drinker have low vitality and feeble resisting powers and pneumonia and Bright's disease are likely to come on any time. There are preventable conditions in every case which can be seen and overcome and their physical character should be recognized. In the periodic drinker, the family physician can do a great deal to prevent the paroxysm by removing causes and correcting abuses. This with psychical and mental treatment succeeds.

The drink attacks resemble epilepsies and can be treated at home. Often institutional treatment is necessary to break up the mental impressions and secure the fullest co-operation of the patient. Then home treatment can be carried on successfully. Other classes of drinking men are anaemic, hyperanaemic, insomniac and suffer from causes which can be corrected and removed, and it is these conditions which the family physician should study.

I am confirmed in the statement that fully 2/3 of all the drinking men in the country should come under medical care and be studied the same as if suffering from any other disease.

The person who at intervals drinks to stupor, distressing his family and destroying himself is a paroxysmal neurotic, clearly is amenable to medical treatment.

He must first be educated to seek health. His family must join him and a physician must know how to give home treatment that will be effective and this treatment must be continued until these paroxysms pass away. This new field of practice must be occupied by the physician to the exclusion of the quack.

The pauper inebriate must come under the same system and be considered diseased, and if incurable housed in hospitals and made to contribute to his own support. There is abundant evidence that these are practical facts, which are calling for a larger and more intelligent recognition of conditions that are now invested in traditions, stupid theories and absolute delusions.

Every spirit and drug neurotic can be restored—many of them permanently by the intelligent co-operation of the family and hospital physician, carried on for a period of years. The physician should study such cases individually and determine how far he can personally treat men with success, and when they should go to hospitals for more exact care and study.

The hospital physician can then send them back for home treatment with a more certain promise of cure, or if the prognosis is bad, permanent hospital restraint in work house hospitals will be called for.

The success of home and office treatment of inebriety is the most promising field of practice and every year my experience confirms the possibility of permanent cures in an increasing number of persons. In all probability the number of neurotics of this class is increasing and the field for their successful treatment is as yet largely unoccupied.

